# Kidney Failure Events, Cardiovascular Disease Events, and All-Cause Mortality in Patients with IgA Nephropathy in a Real-World Database

**DOI:** 10.34067/KID.0000000000000379

**Published:** 2024-02-07

**Authors:** Edgar V. Lerma, Kamlesh M. Thakker, Mark E. Bensink, Richard Lieblich, C. Martin Bunke, Wu Gong, Andrew R. Rava, Kaijun Wang, Diana T. Amari, David Oliveri, Michael V. Murphy, David M.W. Cork, Juan Carlos Q. Velez

**Affiliations:** 1 University of Illinois Chicago/Advocate Christ Medical Center, Oak Lawn, Illinois; 2 Notting Hill Consulting LLC, Celebration, Florida; 3 Travere Therapeutics, Inc., San Diego, California; 4 VJA Consulting, Walnut Creek, California; 5 CM Bunke Consulting, Mount Pleasant, South Carolina; 6 Genesis Research, Hoboken, New Jersey; 7 Genesis Research, Newcastle upon Tyne, United Kingdom; 8 Department of Nephrology, Ochsner Health, New Orleans, Louisiana; 9 Ochsner Clinical School, The University of Queensland, Brisbane, Queensland, Australia

**Keywords:** cardiovascular events, IgA nephropathy, kidney failure, mortality risk, nephrology, proteinuria

## Abstract

**Key Points:**

- In our US real-world cohort study of patients with IgA nephropathy, elevated proteinuria and progression to kidney failure (KF) were associated with a higher risk of cardiovascular disease/mortality events.
- Elevated pre-KF proteinuria was also associated with progression to KF/mortality events.
- Incremental costs associated with CKD stage, nephrotic syndrome, and cardiovascular disease events and of these events were high.

**Background:**

IgA nephropathy (IgAN)–associated glomerular injury leads to proteinuria, hematuria, and progressive loss of GFR, with progression to kidney failure (KF). This retrospective study evaluated the prognostic effects of proteinuria and progression to KF on cardiovascular disease (CVD)/mortality events and KF/mortality events in the United States.

**Methods:**

We conducted a noninterventional, retrospective cohort study in adult patients with IgAN using Optum's deidentified Market Clarity Data (January 1, 2007, to March 31, 2021). Adult (age ≥18 years) patients with at least two signs, disease, symptoms natural language processing term entries for IgAN, within 180 and ≥30 days apart within the identification period were included. Outcomes were assessed by time-dependent proteinuria (≥1 versus <1 g/d) and KF status (pre versus post). Descriptive statistics were used for categorical and continuous variables. Multivariable Cox proportional hazard models with time-dependent predictors were used to estimate differences across groups.

**Results:**

Patients with pre-KF status and proteinuria ≥1 g/d were more likely to have a CVD/mortality event during follow-up (adjusted hazard ratio [HR; 95% confidence interval (CI)]: 1.80 [1.12 to 2.89]; *P* < 0.001) or a KF/mortality event (adjusted HR [95% CI]: 2.10 [1.73 to 2.56]; *P* < 0.001). Patients with post-KF status were more likely to have a CVD/mortality event during follow-up (adjusted HR [95% CI]: 3.28 [2.82 to 3.81]; *P* < 0.001).

**Conclusions:**

Elevated proteinuria and progression to KF were associated with a higher risk of CVD/mortality events. Elevated pre-KF proteinuria was also associated with progression to KF/mortality events. On the basis of our real-world retrospective database analysis, we hypothesize that novel IgAN therapies that reduce proteinuria and slow the rate of progression to KF have the potential to reduce CVD risk, improve kidney outcomes, and prolong/increase overall survival.

## Visual Abstract

**Figure:**
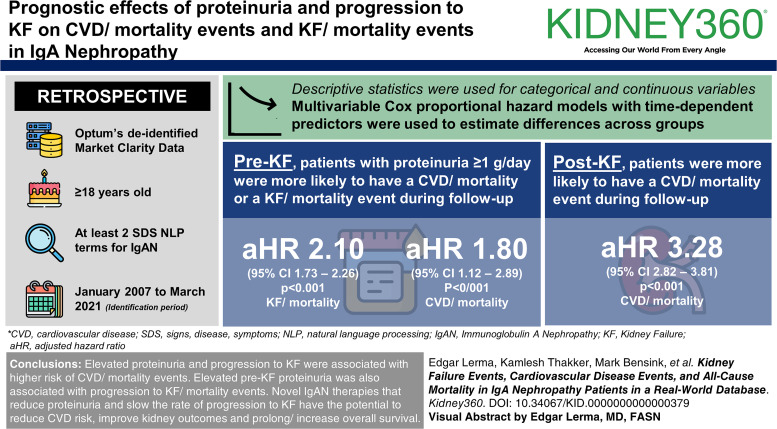

## Introduction

IgA nephropathy (IgAN) is an immune complex–mediated inflammatory disease, which, although rare at the population level, is the most common primary glomerular disease in young non-Hispanic White adults in the United States.^1^ Because of glomerular injury, patients develop proteinuria, hematuria, and progressive loss of GFR, ultimately leading to kidney failure (KF).^2,3^

The Kidney Disease Improving Global Outcomes (KDIGO) guidelines recognize proteinuria ≥0.75–1 g/d as indicative of a high risk of progression and recommend reduction to <1 g/d as a therapeutic goal.^4^ In addition, more advanced CKD stages (eGFR <60 ml/min per 1.73 m^2^) are associated with both cardiovascular and all-cause mortality.^5^ Dynamic assessment of patient risk is recommended to guide treatment decisions, and improved cardiovascular screening in patients with increased proteinuria and high risk of disease progression is critical.^4^

Increasing proteinuria is associated with an increase in cardiovascular risk. The relative risk of cardiovascular death associated with proteinuria ranges from 1.2 to 2.9 on the basis of several population-based cohort studies.^5–7^ However, there is limited research examining this relationship in IgAN. Previous studies were limited in sample size, specificity to IgAN, methods used to capture cardiovascular disease (CVD) events, or inclusion of patients only with advanced disease.^8–14^ However, a recent observational cohort study, from a Canadian registry, reported a crude incidence of CVD events (any major CVD event or revascularization procedure) of 12.2 per 1000 person-years, translating to an absolute 10-year risk of 7.4%.^15^ The standardized incidence ratio for CVD events compared with the general population was 1.38. Proteinuria and lower eGFR were both associated with an increased incidence of CVD events although these associations were not specific to IgAN.^15^ In addition, data from a registry-based analysis of patients with biopsy-proven IgAN suggest that almost all patients with IgAN were expected to progress to KF within their lifetime, regardless of age,^16^ with the implication that all patients are subsequently exposed to more general CKD progression–related adverse cardiovascular outcome risk. Despite this, there remains a need to assess more comprehensively the association of proteinuria reduction and slowing of eGFR decline with CVD events, specifically in IgAN.

In a meta-analysis of 13 IgAN randomized trials, change in proteinuria was found to be associated with KF events.^17,18^ The authors reported a strong and consistent relationship between the level/duration of proteinuria and the decline or loss of kidney function.^17,18^ The severity and duration of sustained proteinuria are commonly assessed by calculating time-averaged proteinuria although the method for calculating time-averaged proteinuria has varied.^19–21^ In our initial investigation into the burden associated with IgAN, we identified that healthcare resource utilization and costs were higher for patients with high-risk proteinuria (≥1 g/d; observed in 54% of the overall and 55% of the adult population at baseline, respectively) and worsening kidney function.^22^

The aim of this study was to evaluate the prognostic effects of proteinuria on the outcome of CVD/all-cause mortality and KF/all-cause mortality using a US real-world data source. In addition, we evaluated the relationship between progression to KF and occurrence of CVD/mortality events. In addition, we completed exploratory analyses of CVD and nephrotic syndrome event rates by proteinuria and post-KF status and the incremental costs associated with nephrotic syndrome events, CVD events, and CKD progression.

## Methods

### Study Design and Data Source

This was an observational, retrospective cohort study using Optum's deidentified Market Clarity Data and proprietary Natural Language Processing (NLP) data.^23^ The Optum deidentified Market Clarity Dataset deterministically links electronic health record data from providers across the care continuum with historical, linked administrative claim data, pharmacy claims, physician claims, and facility claims (with clinical information) and is inclusive of medications prescribed and administered. The Optum NLP system was developed using vocabulary from the Unified Medical Language System that includes multiple medical dictionaries such as the Logical Observation Identifiers Names and Codes, the Systemized Nomenclature of Medicine-Clinical Terms, and RxNorm, a listing of generic and branded drugs (among others). NLP concepts are identified and created based on broad topics such as medications, signs, disease, and symptoms (SDS) terms, measurements, and observations. The data are harvested from the notes fields within the electronic medical records provided to Optum from more than 50 large health care systems throughout the United States. The data used for the development of each NLP concept are deidentified, and accuracy is verified through a series of quality assurance steps before release for use. Each NLP concept included in the data is associated with a unique subject record and a date of observation, allowing longitudinal tracking of concepts over time.

The Optum Market Clarity Dataset is fully Health Insurance Portability and Accountability Act compliant and contains statistician-certified, deidentified data. Institutional Review Board approval was not required for this study.

This study used data between January 1, 2007, and March 31, 2021, with an identification period of July 1, 2007, to March 31, 2021, allowing for a 6-month baseline (Supplemental Figure 1).

### Study Population

**Figure 1:**
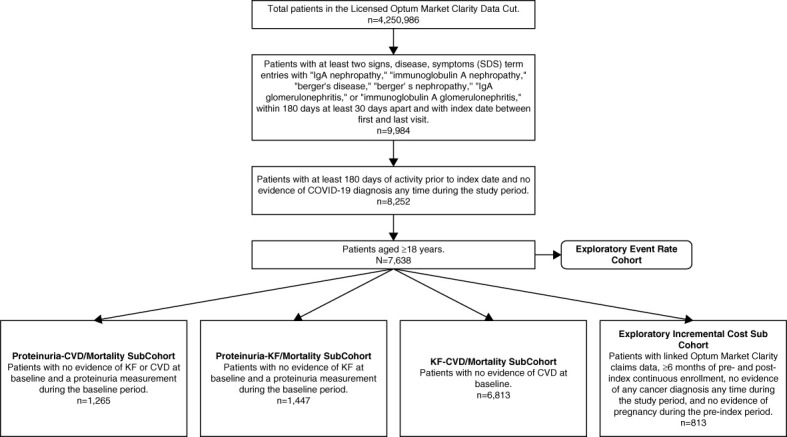
**Cohort attrition**. COVID-19, coronavirus disease 2019; CVD, cardiovascular disease; KF, kidney failure; SDS, signs, disease, and symptoms.

Because of the limited data available on the presence of a kidney biopsy, the study population included adult (age ≥18 years) patients with at least two SDS NLP term entries for “IgA nephropathy,” “immunoglobulin A nephropathy,” “berger's disease,” “berger's nephropathy,” “IgA glomerulonephritis,” or “immunoglobulin A glomerulonephritis,” within 180 and ≥30 days apart within the identification period. Patients with negation terms (*e.g*., “deny,” “failed,” “ignore,” “n/a,” “negative,” “question,” “reject,” “rule out,” “uncertain,” and “unspecified”) in relation to the IgAN SDS term were excluded (Supplemental Figure 2). Patients were required to have ≥6 months of preindex activity (baseline period). The index date was the first IgAN NLP term within the identification period. Patients with evidence of coronavirus disease 2019 before or after index were excluded (Figure 1). Patients were excluded from the proteinuria-CVD/mortality cohort if they had evidence of KF (on the basis of diagnosis codes, eGFR <15 ml/min per 1.73 m^2^, procedure codes for dialysis or kidney transplant) or CVD at baseline (CVD event during baseline period or evidence of myocardial infarction [MI], congestive heart failure, or stroke by the Charlson comorbidity index [CCI]), or if they did not have a proteinuria measurement during the baseline period. For the proteinuria-KF/mortality cohort, patients were excluded if they had evidence of KF at baseline or if they did not have a proteinuria measurement during the baseline period. For the KF-CVD/mortality cohort, patients were excluded if they had evidence of CVD at baseline (CVD event during baseline period or evidence of MI, congestive heart failure, or stroke by the CCI).

### Exposures and Outcomes

Baseline demographics and clinical characteristics included age, sex, region, race/ethnicity (collected by database), insurance type, eGFR, CKD stage, KF, proteinuria, and CCI.

To assess the effect of proteinuria, adjusted hazard ratios (HRs) with 95% confidence intervals (CIs) for CVD/all-cause mortality and KF/all-cause mortality events were reported. Adjusted HRs with 95% CIs were also reported for the association between KF status and CVD/all-cause mortality events.

Proteinuria level was stratified by the KDIGO threshold for high-risk proteinuria (<1 versus ≥1 g/d). To support proteinuria-based analyses, all proteinuria data were presented as g/d with urinary protein-creatinine ratio values in g/g converted to g/d. KF status (pre versus post) was determined based on any of the following: diagnosis codes, eGFR <15 ml/min per 1.73 m^2^, and procedure codes for dialysis or kidney transplant, at any time during follow-up.

CVD events were defined as patients with ≥1 hospital admission with a primary diagnosis of MI, unstable angina, ischemic stroke, transient ischemic attack, or congestive heart failure or ≥1 inpatient or outpatient revascularization procedure (percutaneous coronary intervention, coronary artery bypass graft). Baseline CVD events included those occurring in the 6 months before the index date.

KF events were defined using the same components of the definition for KF status. The first occurrence of the components was considered an event; therefore, patients could only have one occurrence of each component.

All-cause mortality was defined as patients with a death date, calculated as the last day of the death month.

### Statistical Analyses

Baseline demographic and clinical characteristics were analyzed descriptively, including counts and percentages for categorical variables and means with SD and medians with first and third quartiles (Q1–Q3) for continuous variables.

Kaplan–Meier analysis was conducted to assess the association between baseline proteinuria and CVD/mortality or KF/mortality events during follow-up and between baseline KF status and CVD/mortality events during follow-up.

Multivariate Cox proportional hazards models with time-dependent predictors were used to assess the association between proteinuria and CVD/mortality or KF/mortality events, and between KF status and CVD/mortality events. The time-dependent methodology allowed for all time for a given patient to be used in the models: time with proteinuria <1 and/or ≥1 g/d; time with pre-KF and/or post-KF status. Patients with only baseline proteinuria variables were included in the modeling analysis, and their baseline values were used to categorize proteinuria during follow-up.

For the proteinuria and CVD/mortality analysis, censoring occurred at the end of database activity, end of the study period, or transition to post-KF. For the proteinuria and KF/mortality analysis, censoring occurred at the end of database activity or end of the study period. In the KF status and CVD/mortality analysis, censoring occurred at the end of database activity or end of the study period. Full details on the model establishment and variable section are included in the Supplemental Material.

A Cox proportional hazards model adjusting for age, insurance status, sex, diabetes or metastatic solid tumor (CCI), and use of diuretics at baseline was conducted to determine the association between elevated proteinuria and CVD/mortality events in the follow-up period. To determine the association between elevated proteinuria and KF/mortality events in the follow-up period, a Cox proportional hazards model was conducted adjusting for age, insurance status, age/insurance interaction, sex, having a CVD event in the baseline period, baseline CKD stage, liver disease or peptic ulcer disease (CCI), and use of the following medications at baseline: calcineurin inhibitors, mycophenolate, potassium binders, mineralocorticoid receptor antagonists, and renin-angiotensin system inhibitors. For the KF status and CVD/mortality event analysis, a separate Cox proportional hazards model was conducted adjusting for age, insurance status, age/insurance interaction, sex, race/ethnicity, Charlson comorbidities: cancer, liver disease, peripheral vascular disease, diabetes, chronic pulmonary disease, and rheumatologic disease, and use of the following medications at baseline: diuretics, *β* blockers, calcium channel blockers, and renin-angiotensin system inhibitors.

Details on the methods for the exploratory event rate and cost analyses are included in the Supplemental Material.

The data analysis for this study was generated using SAS software. Copyright 2023 SAS Institute Inc. SAS and all other SAS Institute Inc. product or service names are registered trademarks or trademarks of SAS Institute Inc., Cary, NC.

## Results

### Baseline Demographic and Clinical Characteristics

The proteinuria-CVD/mortality cohort included 1265 adult patients: mean age 47 years, 45% female, and 67% non-Hispanic White (Table 1). Median (Q1–Q3) baseline proteinuria was 1.1 (0.3–3.0) g/d; 670 patients had proteinuria ≥1 g/d at baseline. The mean number of proteinuria measurements during follow-up was 4.7, and 19.5% of patients only had a measurement during baseline. The proteinuria-KF/mortality cohort included 1447 adult patients: mean age 48 years, 44% female, and 67% non-Hispanic White (Table 1). The mean number of proteinuria measurements during follow-up was 4.7, and 18.3% of patients only had a measurement during baseline. Median (Q1–Q3) baseline proteinuria was 1.2 (0.3–3.4) g/d; 788 patients had proteinuria ≥1 g/d at baseline. The KF-CVD/mortality cohort included 6813 adult patients: mean age 47 years, 45% female, and 68% non-Hispanic White (Table 1). Among patients with available data, median (Q1–Q3) baseline proteinuria was 1.1 (0.3–3.0) g/d; 1029 patients were post-KF at baseline.

**Table 1:** Baseline patient demographics and clinical characteristics

Characteristics	Proteinuria-CVD/Mortality Cohort (*n*=1265)	Proteinuria-KF/Mortality Cohort (*n*=1447)	KF-CVD/Mortality Cohort (*n*=6813)
**Age, yr**			
Mean (SD)	47 (15)	48 (16)	47 (15)
Median (Q1–Q3)	47 (35–58)	49 (36–60)	47 (36–58)
**Age, yr, *n* (%)**			
18–45	596 (47)	624 (43)	3113 (46)
46–65	514 (41)	588 (41)	2800 (41)
65+	155 (12)	235 (16)	900 (13)
**Sex, *n* (%)**			
Female	566 (45)	633 (44)	3035 (45)
**Region, *n* (%)**			
Midwest	630 (50)	731 (51)	3181 (47)
Northeast	233 (18)	264 (18)	1110 (16)
Other/unknown	42 (3)	50 (3)	267 (4)
South	169 (13)	192 (13)	1136 (17)
West	191 (15)	210 (15)	1119 (16)
**Race/ethnicity, *n* (%)**			
Hispanic (all races)	110 (9)	124 (9)	506 (7)
Non-Hispanic Asian	123 (9)	119 (8)	599 (9)
Non-Hispanic Black	41 (3)	55 (4)	273 (4)
Non-Hispanic White	842 (67)	968 (67)	4600 (68)
Other/unknown	135 (11)	155 (11)	672 (10)
**Insurance type, *n* (%)**			
Commercial	787 (62)	839 (58)	4061 (60)
Medicaid	164 (13)	192 (13)	829 (12)
Medicare	230 (18)	322 (22)	1522 (22)
Other payor type	37 (3)	41 (3)	149 (2)
Uninsured	41 (3)	47 (3)	167 (2)
Unknown	6 (<1)	6 (<1)	85 (1)
**Baseline eGFR, ml/min per 1.73 m** ^**2**^			
With available data, *n* (%)	1239 (98)	1421 (98)	3738 (55)
Mean (SD)	65 (33)	63 (33)	60 (36)
Median (Q1–Q3)	62 (37–91)	58 (34–87)	58 (29–89)
**Baseline CKD stage, *n* (%)**			
With available data	1243 (98)	1425 (98)	4464 (66)
*Stage 1: eGFR >90 or CKD diagnosis*	303 (24)	317 (22)	916 (21)
*Stage 2: eGFR 60–89 or CKD diagnosis*	302 (24)	324 (23)	874 (20)
*Stage 3: eGFR 30–59 or CKD diagnosis*	424 (34)	504 (35)	1158 (26)
*Stage 4: eGFR 15–29 or CKD diagnosis*	214 (17)	280 (20)	555 (12)
*Stage 5: eGFR <15 or CKD diagnosis*	—	—	961 (22)
Unknown	22 (2)	22 (2)	2349 (34)
**Available CKD stage or KF data, *n* (%)**	1243 (98)	1425 (98)	4466 (66)
Baseline KF, *n* (%)	—	—	1029 (23)
Baseline dialysis or renal transplant, *n* (%)	—	—	583 (9)
Stage 5: eGFR <15 or CKD diagnosis	—	—	961 (22)
**Baseline proteinuria, g/d**			
With available data, *n* (%)	1265 (100)	1447 (100)	1265 (19)
Mean (SD)	2.3 (3.4)	2.7 (4.5)	2.3 (3.4)
Median (Q1–Q3)	1.1 (0.3–3.0)	1.2 (0.3–3.4)	1.1 (0.3–3.0)
**CCI**			
Mean (SD)	1.2 (1.3)	1.6 (1.7)	0.8 (1.2)
Median (Q1–Q3)	1.0 (0.0–1.0)	1.0 (0.0–2.0)	1.0 (0.0–1.0)

CCI, Charlson comorbidity index; CVD, cardiovascular disease; KF, kidney failure; Q, quartile.

### Elevated Proteinuria Was Associated With CVD/All-Cause Mortality and KF/All-Cause Mortality Events

**Figure 2:**
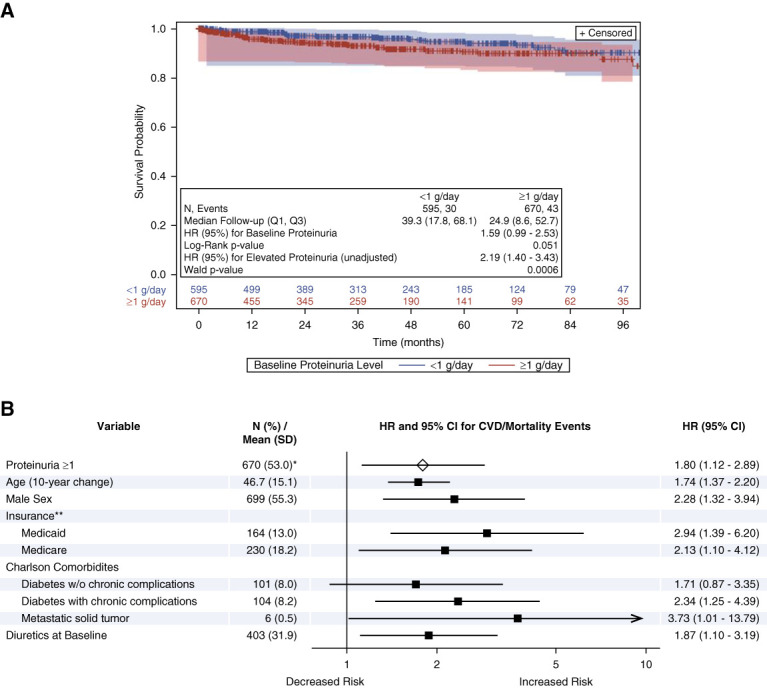
**Elevated proteinuria was associated with CVD/all-cause mortality events.** Kaplan–Meier analysis (A) and Cox proportional model (B) results for proteinuria and CVD/mortality events. Log-rank *P* value from Kaplan–Meier analysis and HR (95% CI) in Kaplan–Meier plot from univariate Cox proportional hazards model. Forest plot generated from the multivariable Cox proportional hazards model. Arrow at the end of the CI bar for metastatic solid tumor indicates the upper end of the CI extends beyond the x axis. *N (%) at baseline. **Reference group=commercial and all other insurance. CI, confidence interval; HR, hazard ratio; Q1, first quartile; Q3, third quartile.

In the proteinuria-CVD/mortality cohort, 30 of 595 patients with baseline proteinuria <1 g/d experienced a CVD/all-cause mortality event over a median (Q1–Q3) follow-up of 39.3 (17.8–68.1) months, while 43 of 670 patients with baseline proteinuria ≥1 g/d experienced a CVD/all-cause mortality event over a median (Q1–Q3) follow-up of 24.9 (8.6–52.7) months. The Kaplan–Meier analysis showed a meaningfully different and borderline significant risk of CVD/mortality for patients with proteinuria >1 g/d at baseline (*P* = 0.05; Figure 2A). In the unadjusted Cox proportional hazards model, proteinuria ≥1 g/d was associated with elevated risk for CVD/mortality events (HR [95% CI]: 2.19 [1.40 to 3.43]; *P* < 0.001). After adjusting for covariates, elevated proteinuria ≥1 g/d remained associated with a significantly elevated risk for CVD/all-cause mortality events (HR [95% CI]: 1.80 [1.12 to 2.89]; *P* < 0.001; Figure 2B).

**Figure 3:**
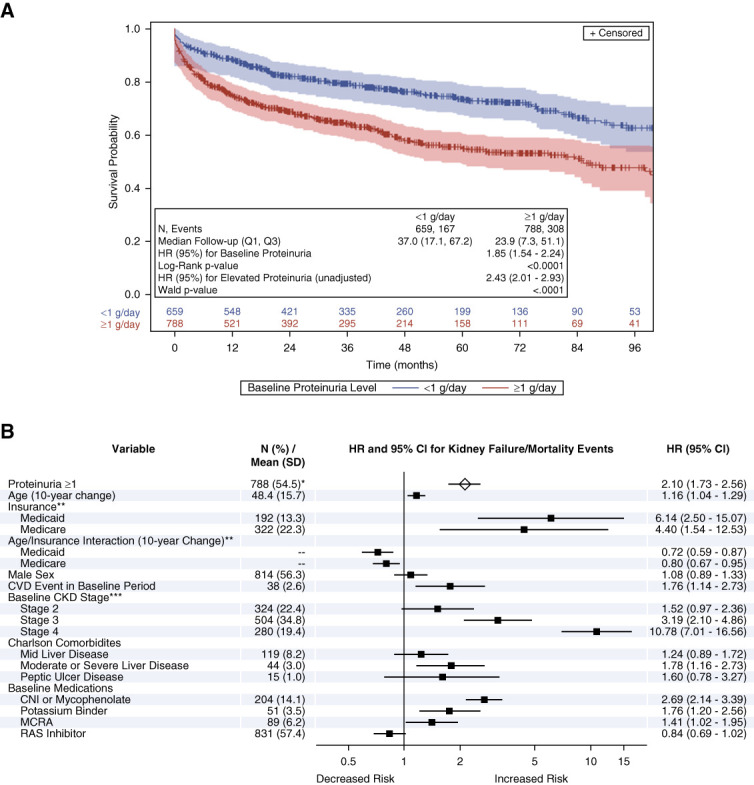
**Elevated proteinuria was associated with KF/All-cause mortality events.** Kaplan–Meier analysis (A) and Cox proportional model (B) results for proteinuria and KF/mortality events. Log-rank *P* value from Kaplan–Meier analysis and HR (95% CI) in Kaplan–Meier plot from univariate Cox proportional hazards model. Forest plot generated from the multivariable Cox proportional hazards model. *N (%) at baseline. Reference groups=**commercial and all other insurance; ***stage 1 and unknown. CNIs, calcineurin inhibitors; MCRA, mineralocorticoid receptor antagonists; RAS, renin-angiotensin system.

In the proteinuria-KF/mortality cohort, 167 of 659 patients with baseline proteinuria <1 g/d experienced a KF/all-cause mortality event over a median (Q1–Q3) follow-up of 37.0 (17.1–67.2) months, while 308 of 788 patients with baseline proteinuria ≥1 g/d experienced a KF/all-cause mortality event over a median (Q1–Q3) follow-up of 23.9 (7.3–51.1) months. The Kaplan–Meier analysis resulted in a significantly higher risk of KF/mortality for patients with proteinuria >1 g/d at baseline (*P* < 0.001; Figure 3A). In the unadjusted Cox proportional hazards model, proteinuria ≥1 g/d was associated with elevated risk for KF/mortality events (HR [95% CI]: 2.43 [2.01 to 2.93]; *P* < 0.001). After adjusting for covariates, proteinuria ≥1 g/d remained associated with a significantly elevated risk for KF/all-cause mortality events (HR [95% CI]: 2.10 [1.73 to 2.56]; *P* < 0.001; Figure 3B).

### KF Status Was Associated With CVD/All-Cause Mortality Events

**Figure 4:**
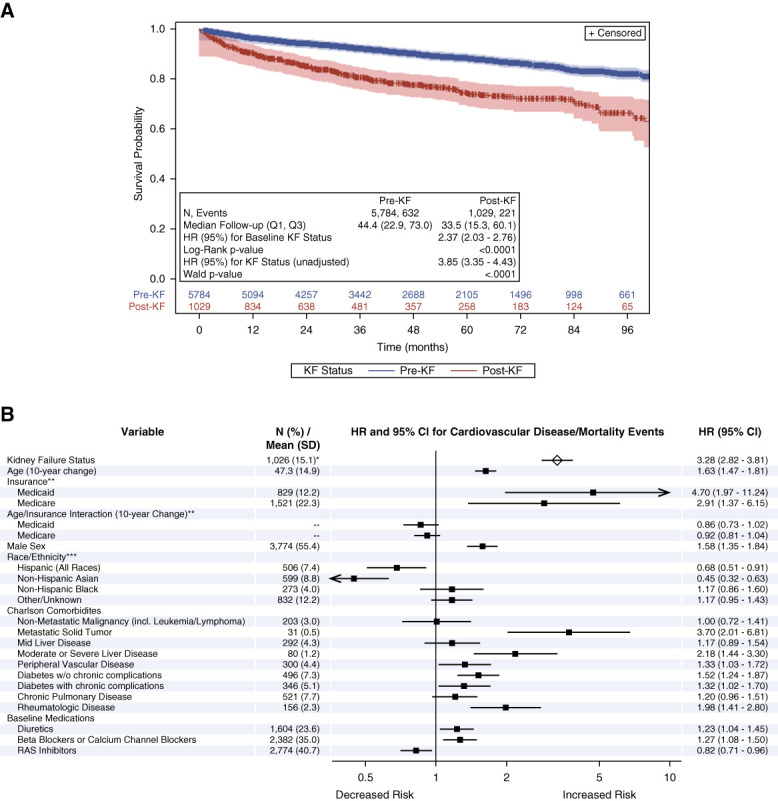
**KF status was associated with CVD/all-cause mortality events.** Kaplan–Meier analysis (A) and Cox proportional model (B) results for KF status and CVD/mortality events. Log-rank *P* value from Kaplan–Meier analysis and HR (95% CI) in Kaplan–Meier plot from univariate Cox proportional hazards model. Forest plot generated from the multivariable Cox proportional hazards model. *N (%) at baseline. Reference groups=**commercial and all other insurance; ***non-Hispanic White.

In the KF-CVD/mortality cohort, 632 of 5784 patients with baseline pre-KF status experienced a CVD/all-cause mortality event over a median (Q1–Q3) follow-up of 44.4 (22.9–73.0) months, while 221 of 1029 patients with baseline post-KF status experienced a CVD/all-cause mortality event over a median (Q1–Q3) follow-up of 33.5 (15.3–60.1) months. The Kaplan–Meier analysis resulted in a significantly higher risk of CVD/mortality for patients with baseline post-KF status (*P* < 0.001; Figure 4A). In the unadjusted Cox proportional hazards model, post-KF status was associated with elevated risk for CVD/mortality events (HR [95% CI]: 3.85 [3.35 to 4.43]; *P* < 0.001). Post-KF status was also associated with elevated risk for CVD/all-cause mortality events (HR [95% CI]: 3.28 [2.82 to 3.81]; *P* < 0.001) after adjustment (Figure 4B).

## Discussion

To our knowledge, this is the first real-world study analyzing the effect of proteinuria on the risk of CVD/mortality events in patients with IgAN in the United States. We demonstrated that elevated proteinuria ≥1 g/d and progression to KF in patients with IgAN were associated with a significantly higher risk of patients experiencing a CVD/mortality event. Our analysis demonstrated consistent results of the prognostic effects of proteinuria through different approaches: the Kaplan–Meier analysis by the baseline proteinuria, the univariate Cox model by time-dependent proteinuria, and the covariate-adjusted Cox model by time-dependent proteinuria. We note that time-dependent proteinuria dynamically reflects disease progression and has been validated as the best metric to account for the prognostic effects of proteinuria over the majority of the follow-up time for IgAN, better than baseline proteinuria and time-averaged proteinuria.^24^ These results highlight the potential for reduction in CVD/mortality risk with effective reduction of proteinuria and prevention of KF in the management of IgAN.

We also showed that pre-KF elevated proteinuria was associated with a significantly elevated risk of progression to KF (defined by diagnosis code, eGFR, dialysis, or transplant status) and/or mortality events. These findings in our US cohort study are consistent with previous observational studies from Canada, Europe, and China, which have demonstrated time-averaged proteinuria to be the strongest predictor of kidney outcomes for patients with IgAN.^19–21^ It should be noted that even among patients with baseline proteinuria <1 g/d in our cohort, 25% of patients progressed to KF, showing similarity with a long-term national registry in the United Kingdom.^15^ These results suggest that a substantial proportion of patients progress to KF despite having proteinuria <1 g/d, the KDIGO recommended threshold for assessing the risk of progression in IgAN.^4^

From our exploratory analyses, CKD stage progression and the occurrence of CVD events were associated with substantial incremental costs although we noted a high level of variability in these results (Supplemental Tables 1-2). The rate of CVD events per 100 person-years was high in patients with elevated baseline proteinuria (≥1 g/d) and for patients with KF at baseline (Supplemental Table 3). Combined, these results align with previous research on the economic burden associated with IgAN^22^ and provide additional insight into the role CVD-related events may play.

Our results must be considered in the context of potential limitations associated with using secondary data sources. For example, this study was limited to patients in the Market Clarity Data and may not be representative of the broader US population. In addition, missing data or errors are inherent in retrospective analyses and errors in detection of IgAN-related terms in patient records may introduce bias. Patients with IgAN-related terms may have more severe disease, the IgAN diagnoses in our cohort were not all biopsy-proven, and MEST-C scores were not available in the dataset. In addition, given limitations in the data, we were unable to identify whether patients were diagnosed with secondary IgAN or IgA vasculitis, which may bias results. It is possible that we have not captured events that occur post-KF because patients transitioned from commercial to traditional Medicare coverage, potentially leading to an underestimation of event rates in this group of patients. The full spectrum of services may not be captured within the dataset, and claims containing enrollment information may have been missed. Within the electronic health record dataset, patients who may have received services in facilities outside of Optum's data would not be captured. Finally, the dataset does not include information on the cause of death; therefore, we were unable to separate non-CVD and non-KF death from our event outcomes.

A clinically meaningful proportion of patients with IgAN experienced CVD events, and our results showed that elevated proteinuria and progression to KF were associated with a statistically higher risk of CVD/mortality events. Elevated pre-KF proteinuria was also associated with progression to KF/mortality events. New IgAN therapies that result in meaningfully greater reductions in proteinuria and slow the rate of eGFR decline may have the potential to reduce CVD risk and economic burden and to improve kidney outcomes and overall survival.

## Supplementary Material

## Data Availability

Data cannot be shared. Partial restrictions to the data and/or materials apply. Deidentified data were used under Travere license agreement with Optum Market Clarity and are not publicly available. Data are available with permission from Optum Market Clarity.

## Supplemental Material

Supplemental Text

Supplemental Figure 1. Study design.

Supplemental Figure 2. Example attrition scenarios for SDS negation terms.

Supplemental Table 1. Baseline patient demographics for the incremental cost subcohort.

Supplemental Table 2. Linear regression analysis of incremental cost associated with CKD stage, nephrotic syndrome events, and cerebrocardiovascular disease (CVD) event.

Supplemental Table 3. Estimated CVD and nephrotic syndrome event rates.

## References

[1] McGrogan A Franssen CF de Vries CS. The incidence of primary glomerulonephritis worldwide: a systematic review of the literature. Nephrol Dial Transplant. 2011;26(2):414–430. doi:10.1093/ndt/gfq665 21068142

[2] Wyatt RJ Julian BA. IgA nephropathy. N Engl J Med. 2013;368(25):2402–2414. doi:10.1056/NEJMra1206793 23782179

[3] Yeo SC Cheung CK Barratt J. New insights into the pathogenesis of IgA nephropathy. Pediatr Nephrol. 2018;33(5):763–777. doi:10.1007/s00467-017-3699-z 28624979 PMC5861174

[4] Kidney Disease Improving Global Outcomes (KDIGO) Glomerular Diseases Work Group. KDIGO 2021 clinical practice guideline for the management of glomerular diseases. Kidney Int. 2021;100(4S):S1–S276. doi:10.1016/j.kint.2021.05.021 34556256

[5] Kannel WB Stampfer MJ Castelli WP Verter J. The prognostic significance of proteinuria: the Framingham study. Am Heart J. 1984;108(5):1347–1352. doi:10.1016/0002-8703(84)90763-4 6496291

[6] Wagner DK Harris T Madans JH. Proteinuria as a biomarker: risk of subsequent morbidity and mortality. Environ Res. 1994;66(2):160–172. doi:10.1006/enrs.1994.1052 8055838

[7] Matsushita K van der Velde M Astor BC, .; Chronic Kidney Disease Prognosis Consortium. Association of estimated glomerular filtration rate and albuminuria with all-cause and cardiovascular mortality in general population cohorts: a collaborative meta-analysis. Lancet. 2010;375(9731):2073–2081. doi:10.1016/S0140-6736(10)60674-5 20483451 PMC3993088

[8] Myllymäki J Syrjänen J Helin H Pasternack A Kattainen A Mustonen J. Vascular diseases and their risk factors in IgA nephropathy. Nephrol Dial Transplant. 2006;21(7):1876–1882. doi:10.1093/ndt/gfl062 16522659

[9] Mahmoodi BK ten Kate MK Waanders F, . High absolute risks and predictors of venous and arterial thromboembolic events in patients with nephrotic syndrome: results from a large retrospective cohort study. Circulation. 2008;117(2):224–230. doi:10.1161/CIRCULATIONAHA.107.716951 18158362

[10] Lee T Derebail VK Kshirsagar AV, . Patients with primary membranous nephropathy are at high risk of cardiovascular events. Kidney Int. 2016;89(5):1111–1118. doi:10.1016/j.kint.2015.12.041 26924046 PMC6787915

[11] Ordoñez JD Hiatt RA Killebrew EJ Fireman BH. The increased risk of coronary heart disease associated with nephrotic syndrome. Kidney Int. 1993;44(3):638–642. doi:10.1038/ki.1993.292 8231039

[12] Heaf J Løkkegaard H Larsen S. The epidemiology and prognosis of glomerulonephritis in Denmark 1985-1997. Nephrol Dial Transplant. 1999;14(8):1889–1897. doi:10.1093/ndt/14.8.1889 10462267

[13] Hutton HL Levin A Gill J Djurdjev O Tang M Barbour SJ. Cardiovascular risk is similar in patients with glomerulonephritis compared to other types of chronic kidney disease: a matched cohort study. BMC Nephrol. 2017;18(1):95. doi:10.1186/s12882-017-0511-z 28320366 PMC5358048

[14] O'Shaughnessy MM Liu S Montez-Rath ME Lafayette RA Winkelmayer WC. Cause of kidney disease and cardiovascular events in a national cohort of US patients with end-stage renal disease on dialysis: a retrospective analysis. Eur Heart J. 2019;40(11):887–898. doi:10.1093/eurheartj/ehy422 30085056

[15] Canney M Gunning HM Zheng Y, . The risk of cardiovascular events in individuals with primary glomerular diseases. Am J Kidney Dis. 2022;80(6):740–750. doi:10.1053/j.ajkd.2022.04.005 35659570

[16] Pitcher D Braddon F Hendry B, . Long-term outcomes in IgA nephropathy. Clin J Am Soc Nephrol. 2023;18(6):727–738. doi:10.2215/CJN.0000000000000135 37055195 PMC10278810

[17] Inker LA Mondal H Greene T, . Early change in urine protein as a surrogate end point in studies of IgA nephropathy: an individual-patient meta-analysis. Am J Kidney Dis. 2016;68(3):392–401. doi:10.1053/j.ajkd.2016.02.042 27032886

[18] Thompson A Carroll K A Inker L, . Proteinuria reduction as a surrogate end point in trials of IgA nephropathy. Clin J Am Soc Nephrol. 2019;14(3):469–481. doi:10.2215/CJN.08600718 30635299 PMC6419287

[19] Reich HN Troyanov S Scholey JW Cattran DC; Toronto Glomerulonephritis Registry. Remission of proteinuria improves prognosis in IgA nephropathy. J Am Soc Nephrol. 2007;18(12):3177–3183. doi:10.1681/ASN.2007050526 17978307

[20] Coppo R Troyanov S Bellur S, . Validation of the Oxford classification of IgA nephropathy in cohorts with different presentations and treatments. Kidney Int. 2014;86(4):828–836. doi:10.1038/ki.2014.63 24694989 PMC4184028

[21] Le W Liang S Hu Y, . Long-term renal survival and related risk factors in patients with IgA nephropathy: results from a cohort of 1155 cases in a Chinese adult population. Nephrol Dial Transplant. 2012;27(4):1479–1485. doi:10.1093/ndt/gfr527 21965586

[22] Lerma EV Bensink ME Thakker KM, . Impact of proteinuria and kidney function decline on Healthcare costs and resource utilization in adults with IgA nephropathy in the United States: a retrospective analysis. Kidney Med. 2023;5(9):100693. doi:10.1016/j.xkme.2023.100693 37637862 PMC10457441

[23] Optum Market Clarity Data Product Sheet. 2020. Accessed July 4, 2023. https://www.optum.com/content/dam/optum3/optum/en/resources/brochures/market-clarity-data-product-sheet.pdf

[24] Barbour SJ Cattran DC Espino-Hernandez G Hladunewich MA Reich HN. Identifying the ideal metric of proteinuria as a predictor of renal outcome in idiopathic glomerulonephritis. Kidney Int. 2015;88(6):1392–1401. doi:10.1038/ki.2015.241 26287314

