# Supplementary material for: Kidney Failure Events, Cardiovascular Disease Events, and All-Cause Mortality in Patients with IgA Nephropathy in a Real-World Database
Source: Kidney360. 2024 Feb 7;5(3):427–36. doi: 10.34067/KID.0000000000000379 (PMC11000736; doi:10.34067/KID.0000000000000379)
Supplement: Supplementary file 1 [file kidney360-5-427-s001.pdf]

Supplemental material

Supplemental Text

Supplemental Figure 1 – Study Design

Supplemental Figure 2 – Example Attrition Scenarios for SDS negation terms

Supplemental Table 1 – Baseline Patient Demographics for the Incremental Cost Sub-Cohort

Supplemental Table 2 – Linear Regression Analysis of Incremental Cost Associated with CKD Stage, Nephrotic Syndrome Events and Cerebrocardiovascular disease (CVD) Event

Supplemental Table 3 – Estimated CVD and Nephrotic Syndrome Event Rates

Supplemental Text

Model Establishment Methodology

Univariate associations for all demographic (age, gender, region, insurance type, index year, age/insurance interaction), baseline health status (Charlson comorbidities, Charlson comorbidity index, CVD events, CKD stage) and baseline medication (antihypertensives, beta blockers, calcium channel blockers, mineralocorticoid receptor antagonists, immunosuppressives, calcineurin inhibitors, renin-angiotensin system inhibitors, potassium binders, mycophenolate, diuretics, statins, angiotensin-converting enzyme inhibitors, angiotensin II receptor blockers, sodium-glucose cotransporter-2 inhibitors, glucagon-like peptid-1 receptor antagonists, rituximab, and glucocorticoids) variables were tested for inclusion in the multivariable model. Variables with a p-value <0.30 were considered for inclusion in the multivariable model. The multivariable models were established by initially including the independent variable of interest only. Variables were added to the model in a stepwise fashion with variables retained in the model if they were statistically significant (p <0.05) or if their addition improved the model's Akaike information criterion (AIC) or had an appreciable impact on the HR for the independent variable of interest. If the addition of a new variable caused a previously retained variable to lose statistical

significance, we tested removing the variable and assessed model AIC and HR for the variable of interest. The proportional hazards assumption was not tested as the violation occurred at a late point (~72 months) in the analysis where a very selective subgroup of patients with sufficient follow-up remained.

Exploratory Incremental Costs Analysis

The identification period for the exploratory incremental cost sub-cohort was July 1, 2007 to September 30, 2020, allowing for a 6 month follow-up (**Supplemental Figure 1)**. For inclusion in the exploratory incremental cost sub-cohort, patients were required to have linked Optum® Market Clarity claims data, and ≥6 months of pre- and post-index continuous enrollment. Those with evidence of cancer or COVID-19 pre- or post-index, or pregnancy pre-index were excluded (**Figure 1**).

The incremental costs associated with having a CVD or KF event were estimated using a linear regression model, adjusting for age, Charlson Comorbidity Index (CCI), baseline CKD stage, gender, index year, insurance type, region, proteinuria events, steroid use events, and nephrotic-level proteinuria events. The log-transformed costs were modeled using a Gaussian distribution and identity link. Costs were re-transformed using a smear technique. All costs were adjusted to 2020 US dollars using the Consumer Price Index and presented as per-patient-per-month (PPPM) values.

Exploratory Event Rate Analysis

The exploratory event rate study used data between January 1, 2007 and March 31, 2021 with an identification period of July 1, 2007 to March 31, 2021, allowing for a 6 month baseline (**Supplemental Figure 1**). The study population included adult (≥18 years old) patients with at least two SDS NLP term entries for “iga nephropathy", "immunoglobulin A nephropathy", "berger's disease", "berger’s nephropathy", "iga glomerulonephritis", or "immunoglobulin A glomerulonephritis", within 180 days and ≥30 days apart within the identification period. Patients with negation terms (e.g., 'deny', 'failed', 'ignore', 'n/a', 'negative', 'question', 'reject', 'rule out', 'uncertain', 'unspecified') in relation to the IgAN SDS term were excluded. Patients were required to have ≥6 months of pre-index activity (baseline period). The

index date was the first IgAN NLP term within the identification period. Patients with evidence of COVID-19 pre- or post-index were excluded (**Figure 1**).

Rates of CVD and nephrotic syndrome events were reported as events per 100 person years following the index date. Patients were followed from the index date to the end of EHR/claims activity or to the end date of study period. End of follow-up was the earliest of the end date of EHR/claims activity or end date of study period (March 31, 2021). CVD events were defined as patients with ≥1 hospital admission with primary diagnosis of myocardial infarction (MI), unstable angina, ischemic stroke, transient ischemic attack (TIA), or congestive heart failure or ≥1 inpatient or outpatient revascularization procedure (percutaneous coronary intervention [PCI], coronary artery bypass graft [CABG]). Nephrotic syndrome events were defined as patients with ≥1 protein-creatinine ratio≥3.0 g/g or 24-hour urine protein ≥3.5 g/day and serum albumin levels <3.0 g/dL or with ≥1 hospital admission or outpatient visit with diagnosis associated with nephrotic syndrome.

Supplemental Figure 1: Study Design

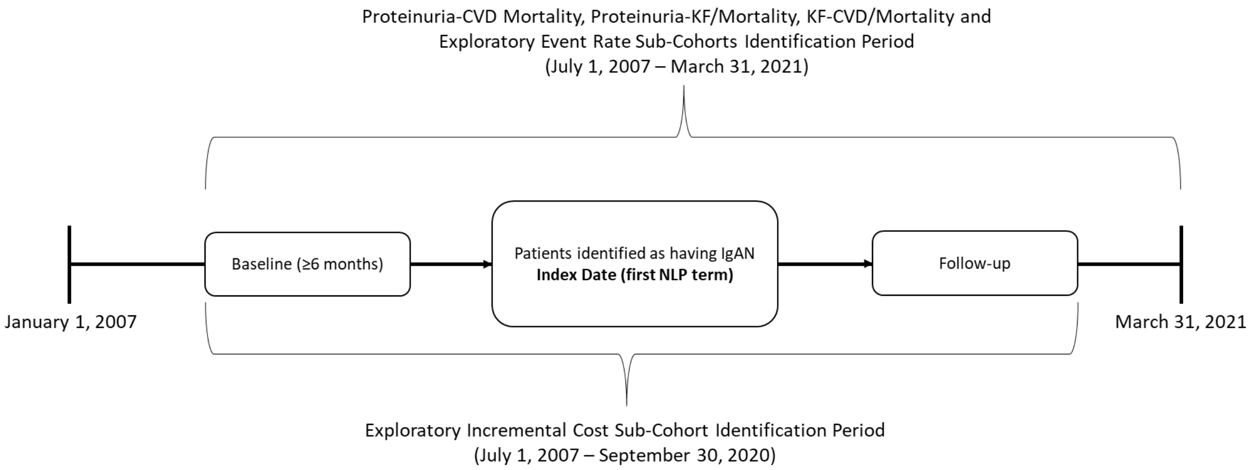

Abbreviations: CVD, cardiovascular disease; IgAN, IgA nephropathy; KF, kidney failure; NLP, natural language processed

Supplemental Figure 2: Example Attrition Scenarios for SDS negation terms

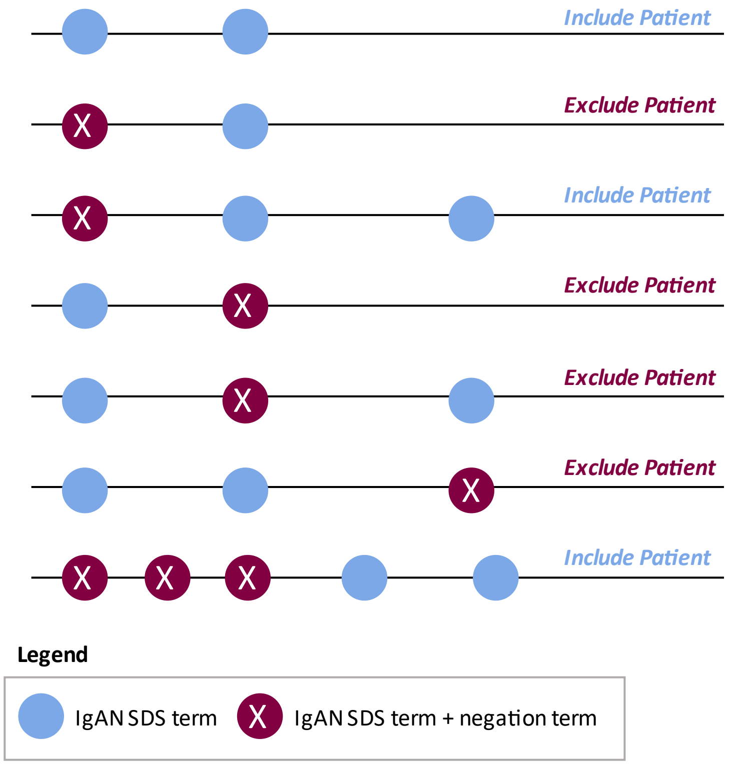

*Assuming eligible SDS terms are 30 days apart, within 180 days

Abbreviations: IgAN: immunoglobulin A nephropathy; SDS: signs, disease and symptoms

Supplemental Table 1: Baseline Patient Demographics and Clinical Characteristics for the Exploratory Incremental Cost Sub-Cohort

**Characteristics**	**Exploratory Incremental Cost Sub-Cohort (n=813)**
**Age, years**	
Mean (SD)	47 (14)
Median (Q1-Q3)	48 (38 – 57)
**Age, n (%)**	
18-45 years	350 (43%)
46-65 years	393 (48%)
65+ years	70 (9%)
**Gender, n (%)**	
Female	303 (37%)
**Region, n (%)**	
Midwest	326 (40%)
Northeast	208 (26%)
Other/Unknown	34 (4%)
South	124 (15%)
West	121 (15%)
**Race/Ethnicity, n (%)**	
Hispanic (All Races)	57 (7%)
Non-Hispanic Asian	65 (8%)
Non-Hispanic Black	29 (4%)
Non-Hispanic White	540 (66%)
Other/Unknown	122 (15%)
**Insurance Type, n (%)**	
Commercial	554 (68%)
Medicaid	92 (11%)
Medicare	146 (18%)
Other Payor Type	2 (<1%)
Uninsured	9 (1%)
Unknown	10 (1%)
**Baseline eGFR, mL/min/1.73 m^2**	
With available data**, n (%)**	448 (55%)
Mean (SD)	58 (35)
Median (Q1-Q3)	57 (29 – 87)
**Baseline CKD stage, n (%)**	
With available data	584 (72%)
Stage 1: eGFR >90 or CKD diagnosis	104 (18%)
Stage 2: eGFR 60-89 or CKD diagnosis	108 (18%)
Stage 3: eGFR 30-59 or CKD diagnosis	167 (29%)
Stage 4: eGFR 15-29 or CKD diagnosis	77 (13%)
Stage 5: eGFR <15 or CKD diagnosis	128 (22%)
Unknown	229 (28%)
**Available CKD stage or KF data, n (%)**	584 (72%)
Baseline KF, n (%)	138 (24%)
Baseline dialysis or renal transplant, n (%)	101 (17%)
Stage 5: eGFR <15 or CKD diagnosis, n (%)	128 (22%)
**Baseline proteinuria, g/day**	
With available data, n (%)	167 (21%)
Mean (SD)	2.9 (5.3)
Median (Q1-Q3)	1.5 (0.4 – 3.3)

**CCI**	
Mean (SD)	1.2 (1.5)
Median (Q1-Q3)	1.0 (0.0 – 1.0)

Abbreviations: CCI, Charlson comorbidity index; CKD, chronic kidney disease; CVD, cardiovascular disease; eGFR, estimated glomerular filtration rate; KF, kidney failure; Q, quartile; SD, standard deviation

Supplemental Table 2: Linear Regression Analysis of Incremental Cost Associated with CKD Stage, Nephrotic Syndrome Events and Cerebrocardiovascular disease (CVD) Event

**Event**	**Incremental Cost (PPPM)**	**SD**
CKD Stage 3	$1,081.75	$27,397.30
CKD Stage 4	$1,822.19	$44,812.20
CKD Stage 5	$7,341.65	$88,809.90
Nephrotic Syndrome Event	$1,268.04	$39,130.10
CVD Event	$6,041.45	$81,113.00

Incremental cost by CKD stage is based on average cost for CKD 1/2; $1788.43
Adjusted for age, Charlson comorbidity index, baseline CKD stage, gender, index year, insurance type, region, proteinuria events, steroid use events, and nephrotic-level proteinuria events

Abbreviations: CI, confidence interval; CKD, chronic kidney disease; CVD, cardiovascular disease; PPPM, per-patient-per-month

Supplemental Table 3: Estimated CVD and Nephrotic Syndrome Event Rates

	**Event Type**	**Patients**	**Events**	**Person Years**	**Rate (per 100 PY)**	**95% CI**
**Baseline proteinuria <1 g/day**	CVD event	671	166	2991	5.5	4.8-6.5
	Nephrotic Syndrome event	671	185	2991	6.2	5.4-7.1
**Baseline proteinuria ≥1 g/day**	CVD event	776	339	3079	11.0	9.9-12.2
	Nephrotic Syndrome event	776	985	3079	32.0	30.1-34.1
**Post-KF**	CVD event	3188	1,506	11669	12.9	12.3-13.6

Abbreviations: CI, confidence interval; CVD, cardiovascular disease; KF, kidney failure; PY, person-years
